# Halogenated flame retardant exposure was associated with pediatric gut microbiome profiles in a Canadian cohort

**DOI:** 10.1080/29933935.2026.2747517

**Published:** 2026-10-18

**Authors:** Yidan Zhang, Feng Gao, Peng Gao, Yihan Fan, Xinlei Wang, Yuqiu Yang, Anthony Gagnon, Guillaume Martinez, Virginie Gillet, Jiping Zhu, Jean-Philippe Bellenger, Andrea A. Baccarelli, Larissa Takser, Yike Shen

**Affiliations:** a Department of Earth and Environmental Sciences, University of Texas at Arlington, Arlington, TX, USA; b Department of Environmental Health Sciences, Fielding School of Public Health, University of California Los Angeles (UCLA), Los Angeles, CA, USA; c Department of Molecular and Medical Pharmacology, David Geffen School of Medicine, University of California, Los Angeles (UCLA), Los Angeles, CA, USA; d Department of Environmental Health and Department of Molecular Metabolism, Harvard T.H. Chan School of Public Health, Boston, MA, USA; e Department of Mathematics, University of Texas at Arlington, Arlington, TX, USA; f Quantitative Biomedical Research Center, Department of Health Data Science and Biostatistics, Peter O’Donnell Jr. School of Public Health, University of Texas Southwestern Medical Center, Dallas, TX, USA; g Département de Pédiatrie, Faculté de Médecine et des Sciences de la Santé, Université de Sherbrooke, Sherbrooke, QC, Canada; h Département de Chimie, Université de Sherbrooke, Sherbrooke, QC, Canada; i Environmental Health Science and Research Bureau, Health Canada, Ottawa, ON, Canada; j Department of Environmental Health, Harvard T.H. Chan School of Public Health, Boston, MA, USA

**Keywords:** Halogenated flame retardants, pediatric gut microbiome, metagenome, metatranscriptome

## Abstract

Environmental chemical exposures have been linked to gut microbiome dysregulation and adverse health outcomes in children. Halogenated flame retardants (HFRs) are persistent chemicals with substantial early-life exposure, yet evidence on their effects on the pediatric gut microbiome remains limited. We examined associations between HFR exposures and the gut microbiome among 8–12-y-old children from the GESTation and Environment (GESTE) cohort in Sherbrooke, Canada. In a multi-matrix, multi-omics design, 10 HFRs were quantified in plasma (*n* = 140) and stool (*n* = 182), with shotgun metagenomic and metatranscriptomic sequencing characterized microbial species and functional pathways. After false discovery rate correction, plasma BDE-47 and BDE-99 were inversely associated with *Clostridium sp. CAG:221* and *Bacteroides dorei*, respectively (*q* < 0.2). Stool BDE-153 was negatively associated with short-chain fatty acid–producing taxa, including *Eubacterium spp.* and *Roseburia intestinalis*, and positively associated with *Alistipes putredinis* and *Faecalibacterium spp*. Stool BDE-153 and anti-DP were associated with higher activity of pathways involved in phospholipid homeostasis and 4-aminobutanoate degradation. Mediation analysis suggested that *Faecalibacterium spp*. partly mediated the association between stool BDE-153 and cytidine diphosphate-diacylglycerol biosynthesis. Our findings suggest that childhood HFR exposure is associated with alterations in gut microbiome composition and function, with potential implications for pediatric health.

## Graphical abstract

**Figure:**
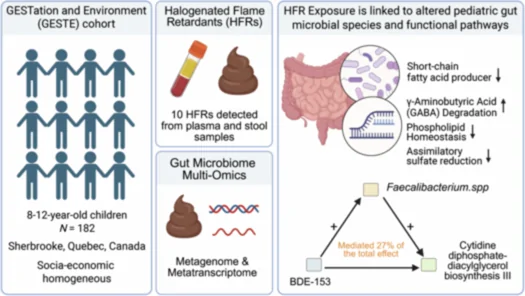

## Introduction

Halogenated flame retardants (HFRs) are a group of synthetic semi-volatile chemicals containing bromine or chlorine atoms and are widely used to reduce flammability in daily-use products such as furniture, electronics, textiles, building materials, and plastics.^1^ ^,^ ^2^ Because HFRs are typically added rather than chemically bound to materials, they can leach, volatilize, or degrade into the surrounding environment, leading to widespread human exposure through inhalation, ingestion, or dermal contact.^3^ Toxicological studies indicated that both brominated flame retardants (e.g., polybrominated diphenyl ethers, PBDEs) and chlorinated compounds (e.g., Dechlorane Plus, DP) can disrupt multiple biological systems, including the endocrine, immune, and nervous systems.^4‐7^ More recently, growing attention has turned toward the gut microbiome as a potential novel target of HFR toxicity, given its crucial role in host metabolism, immunity, and development.^8‐12^

The gut microbiome is a complex and dynamic microbial community that is essential for human health, contributing to nutrient absorption, metabolism, immune development, and neuroendocrine signaling.^13‐15^ Dysbiosis of gut microbiomes has been linked to various diseases, including metabolic disorders, neurological diseases, digestive symptoms, and cancers.^13^ Human exposure to HFRs occurs through multiple pathways, including ingestion and inhalation of indoor dust and consumption of contaminated food, especially animal-derived and lipid-rich foods such as fish/seafood, meat, and dairy products.^16‐18^ After ingestion, HFRs may be poorly absorbed and thus transported to the distal intestine, or absorbed compounds may later partition back from the circulation into the gut, where they can directly interact with the gut microbiome.^19^ In this process, children are more vulnerable due to their hand-to-mouth behaviors, greater dust intake relative to body weight, and an immature digestive system.^17,20‐22^ Although mechanisms underlying HFR-gut microbiome interactions are not fully elucidated, several biological pathways have been proposed. First, HFRs may activate host nuclear receptors, such as pregnane X and constitutive androstane receptor altering microbial survival conditions.^23^ Second, experimental evidence indicates that HFR can disrupt bile acid and lipid metabolism, reshaping the gut microbial environment.^24^ ^,^ ^25^ Third, HFRs in the gut may impair intestinal epithelial barrier integrity, increase intestinal permeability, and promote dysbiosis.^26^ Together, these mechanisms suggest that HFRs may influence both the composition and function of the gut microbiome.

Evidence on the associations between HFR exposures and human gut microbiome profiles is growing but still sparse. Several in vivo and in vitro studies have demonstrated alterations in microbial composition and metabolite profiles following PBDEs and DP exposure.^9‐12,27‐30^ Human studies are more limited, with only two prior studies examining the associations between HFR exposures and the human gut microbiome. A Norwegian birth cohort examined associations between six PBDEs in breast milk and infant gut microbiome assessed by 16S rRNA sequencing.^31^ They reported that higher BDE-28 concentrations in breast milk were associated with lower microbial diversity and reduced short-chain fatty acid production in infants.^31^ Similarly, in a pilot study within the Canadian GESTation and Environment (GESTE) cohort, associations between four PBDEs measured in maternal plasma and the gut microbiome of offspring aged 6–8 years, assessed by 16S rRNA sequencing, were examined.^32^ The study reported that BDE-47 (2,2′,4,4′-tetrabromodiphenyl ether) and BDE-99 (2,2′,4,4′,5-pentabromodiphenyl ether) were associated with lower abundances of bacteria in the *Ruminococcaceae* family and the *Ruminococcus_2* genus in children’s gut microbiomes.^32^ Despite existing evidence, important research gaps remain. First, existing studies have focused predominantly on PBDEs,^31^ ^,^ ^32^ whereas evidence for other widely used HFRs, such as chlorinated flame retardants, remains scarce despite their growing global use. Second, prior research has largely relied on circulating biomatrices (e.g., blood and breast milk),^31^ ^,^ ^32^ while stool HFR measurements remain understudied. HFR concentrations measured in different biological matrices reflected distinct aspects of exposure and toxicokinetic. Previous studies have shown that lower-brominated PBDEs are more likely to be retained in body tissues,^33^ ^,^ ^34^ with estimated adult blood half-lives of 1.4–2.4 years for BDE-47 and 3.6–12.4 years for BDE-153.^35^ In contrast, highly brominated PBDEs such as BDE-209 and some chlorinated flame retardants, have shorter apparent blood half-lives (i.e, 11–18 days) and substantial fecal excretion. ^36^ ^,^ ^37^ These differences suggest that blood and stool measurements may provide complementary information on HFR exposure. Moreover, HFRs present in the intestinal lumen may directly interact with gut microorganisms, highlighting the relevance of examining stool HFR concentrations in gut microbiome research. Third, 16S rRNA sequencing had been mostly used in previous studies,^31^ ^,^ ^32^ providing limited taxonomic resolution and functional insight, whereas shotgun metagenomics and metatranscriptomics could better reveal species-level profiles and active microbial functions linked to HFR exposure.

This study aims to address these research gaps by investigating associations of both legacy and emerging HFRs measured in plasma and stool with gut microbial taxa and functional profiles in a homogenous socioeconomic status cohort of school-aged children. We hypothesized that childhood HFR exposure was associated with alterations in gut microbial composition and functional pathways, and that associations between HFR exposure and microbial functions would be partially explained by variation in specific gut microbial taxa. By quantifying HFR concentrations in both plasma and stool and characterizing gut microbial species and active functional pathways using shotgun metagenomic and metatranscriptomic sequencing, this study provides a comprehensive assessment of how HFR exposure might associated with the composition and function of children's gut microbiome.

## Method

### Study population

This study was conducted in the GESTation and Environment (GESTE) cohort in Sherbrooke, Quebec, Canada. The participants in the GESTE cohort are homogeneous in socio-economic status and race/ethnicity, with most self-identifying as middle-class, White, and French–Canadian. Women were recruited into the GESTE cohort between 2007 and 2009 during the first trimester of pregnancy or at delivery (*n* = 800), including 400 mothers recruited during pregnancy (mean gestational age = 12 weeks, SD = 2.96 weeks) and 400 at delivery. Eligibility criteria included maternal age ≥18 y, gestational age <20 weeks for those recruited during pregnancy, and no known thyroid disease. Women reporting illicit drug use (*n* = 8) were excluded.^38^ ^,^ ^39^ At follow-up, 310 children were followed up at age 8–12 y. Among these, 193 provided stool samples and 172 provided blood samples for HFR measurement. For gut microbiome profiling, stool samples were available from 247 children for metagenomic sequencing and from 239 children for metatranscriptomic sequencing. The sample sizes and schematic workflow of our study is shown in Figure 1. The complete-case analysis sets ranged from 122 to 182 participants.

Parents provided informed and written consent at each follow-up. All study protocols were approved by the institutional review boards of the Research Center of the Center Hospitalier Université de Sherbrooke (CHUS) and Harvard T. H. Chan School of Public Health.

**Figure 1.**
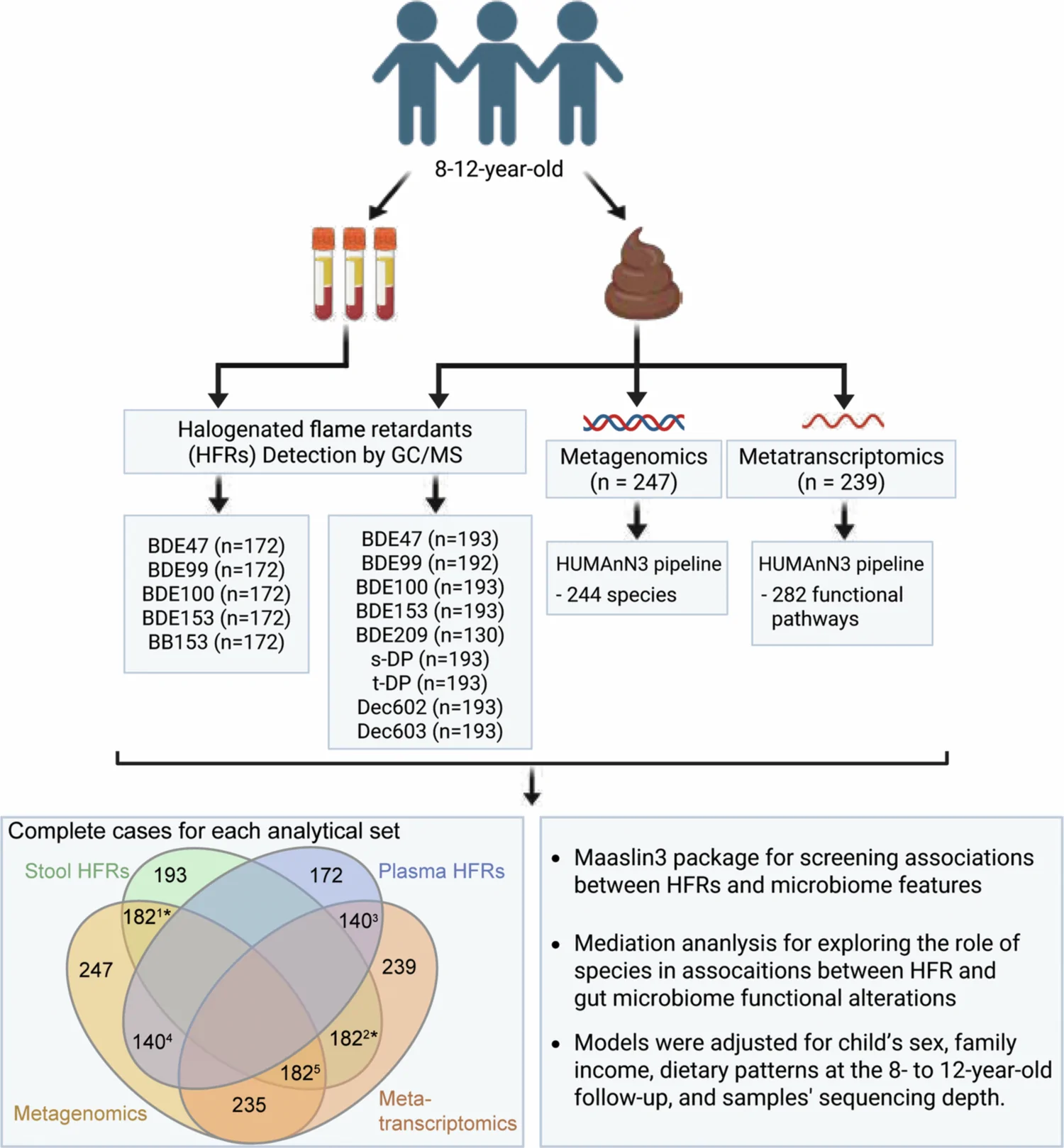
Schematic of the study workflow in the GESTation and Environment (GESTE) cohort in Sherbrooke, Quebec, Canada.^1,2^ Complete cases between stool HFRs and metagenomic data were 182.^3,4^ Complete cases between plasma HFRs and metatranscriptomic data were 140.^5^ Complete cases with stool HFRs, metagenomic, and metatranscriptomic data were 182. *The complete cases with stool BDE-99 and metagenomic/metatranscriptomic data were 181, and the complete cases with stool BDE-209 and metagenomic/metatranscriptomic data were 122.

### Sample collection

Blood and stool samples were collected from children. Blood draws were conducted at the Center Hospitalier Universitaire de Sherbrooke (CHUS) from 2019 to 2022. Samples were collected in 10 mL sodium heparin tubes, centrifuged at 1500 × g for 15 minutes at 4 °C, and 2 mL of plasma was transferred to glass vials and stored at −20 °C until analysis. Stool samples were collected from children during the 8–12–y follow-up either at home or at the research center of the Center Hospitalier Universitaire de Sherbrooke (CHUS). Children were assisted by their parents during collection. Briefly, stool was collected using a compostable receptacle placed in the toilet, and parents transferred portions of the sample into separate collection vials while wearing gloves. One aliquot was immediately placed in a vial containing RNAlater for metatranscriptomic analyzes, while another two aliquots were collected for metagenomic and HFR analyzes. After collection, samples were temporarily stored in household freezers at −20 °C for up to 48 hours before transport to the research center. Stool samples for metagenomic and metatranscriptomic sequencing were subsequently stored at −80 °C until analysis, whereas separate aliquots for HFR analyzes were stored at −20 °C. Further details on the collection procedures are available in previous publications.^40^ ^,^ ^41^

### Exposure assessment

We quantified 10 HFRs, including legacy PBDEs, bromobiphenyls, and emerging chlorinated flame retardants, in both stool and plasma samples. Target analytes included six PBDE congeners, BDE-47, BDE-99, BDE-100 (2,2',4,4',6-pentabromodiphenyl ether), BDE-153 (2,2',4,4',5,5'-hexabromodiphenyl ether), BDE-209 (decabromodiphenyl ether), as well as BB-153 (2,2',4,4',5,5'-hexabromobiphenyl). Emerging HFRs included syn- and anti-Dechlorane Plus (stereoisomers of 1,1'-(1,4-ethanediyl) bis [2,3,4,5,6-pentachlorobenzene]), Dechlorane (Dec)-602, and Dec603. All HFR concentrations were quantified using gas chromatography–mass spectrometry at the Département de Chimie, Université de Sherbrooke. Five compounds in plasma (BDE-47, BDE-99, BDE-100, BDE-153, BB-153) and nine in stool (BDE-47, BDE-99, BDE-100, BDE-153, BDE-209, anti-DP, syn-DP, Dec602, Dec603) were detected in >30% of participants and included in the association analyzes. Total plasma lipid content (g/L) was assessed using previously described methods.^42^ Briefly, plasma samples (150 mL) were thawed on ice and sonicated for 30 minutes. Total cholesterol (TC) and triglycerides (TG) were measured using an enzymatic colorimetric method on a Roche Cobas 8000 analyzer. Wet-weight plasma concentrations (ng/mL) were converted to lipid-normalized values by multiplying by 1000 and adjusting for the measured lipid content. Stool sample concentrations were standardized to dry weight by dividing the quantified mass of each compound by the dry mass of the corresponding stool specimen, yielding concentrations expressed as ng per gram dry weight (ng/g dw). Values below the limit of detection (LOD) were imputed as LOD divided by $\sqrt{2}$ . Detailed analytical procedures have been previously described.^43^

### DNA/RNA extraction and multi-omics profiling

Stool samples were processed for both metagenomic and metatranscriptomic analyzes as previously described.^41^ Default parameters were used for all bioinformatics tools. For metagenomic data processing, raw sequencing reads underwent quality control, including adapter trimming and quality filtering using Trimmomatic (v0.39). Host-derived reads were removed by alignment to the human reference genome (hg38) using Bowtie2 (version 2.5.4), retaining unaligned paired-end reads. The mean ± SD sequencing depth after host read removal was 35.3 ± 27.4 million reads per sample. Detailed read count statistics across sequencing quality-control steps are summarized in Figure S1. Taxonomic profiling was performed using MetaPhlAn (v3.0) with the ChocoPhlAn database (mpa_v31_CHOCOPhlAn_201901).^44^ Species detected in fewer than 10% of samples were excluded, leaving 244 species for downstream analysis.

For metatranscriptomics sequencing, RNA was extracted using the RNeasy PowerMicrobiome kit, assessed via Bioanalyzer, depleted of rRNA (Ribo-Zero Plus), and sequenced using the same platform with metagenomic sequencing. Raw reads were processed with KneadData (v0.12.0) to remove host and rRNA sequences. The mean ± SD shotgun metatranscriptome sequencing depth after removing host and rRNA sequences was 34.0 ± 31.5 million reads per sample. Functional annotation was performed using HUMAnN (v3.0) with the UniRef90 database (UniRef90_201901b), with translated protein alignments conducted using DIAMOND (v2.1.10). Pathways present in fewer than 10% of samples were removed, resulting in 282 expressed pathways for analysis.

Metagenomic and metatranscriptomic samples were processed and sequenced in two batches, including 115 and 67 samples for metagenomics and 118 and 64 samples for metatranscriptomics in the first and second batches, respectively. Potential batch effects were evaluated using principal component analysis (PCA) at the species and pathway levels. While no clear separation by sequencing batch was observed at the species level, PCA revealed batch-related separation in the metatranscriptomic functional pathway profiles (Figure S2). A subsequent PERMANOVA analysis confirmed significant differences in pathway profiles between the two sequencing batches (R^2^ = 0.27, *p* = 0.001). Quality control procedures included blank control samples and four technical duplicate samples. Blank control samples yielded extremely low read counts and did not produce usable sequencing profiles. Technical duplicate samples were used for quality assessment purposes only and were excluded from downstream analyzes.

### Covariates

Covariates were selected if they simultaneously met the following criteria: they were well-established predictors of the childhood gut microbiome, determinants of HFR exposure, and not mediators on the causal pathway from HFR exposure to the gut microbiome.^45^ The following covariates were retained in the final models: sex (male vs. female),^46^ ^,^ ^47^ family income at the 8- to 12-y-old follow-up (continuous variable, in Canadian dollars per year),^48^ ^,^ ^49^ parents' educational level (secondary, vocational training, college, and university),^48^ ^,^ ^50^ and dietary patterns (meat and snack-rich pattern, dairy-rich pattern, and seafood-rich pattern).^18^ ^,^ ^51^ Sample's metagenomic and metatranscriptomic sequencing depth were also adjusted in the model to control for technical variation that could bias microbial abundance estimates. Data on sex, family income, and parents' educational level were obtained from questionnaires at the 8- to 12-y-old follow-up stage. Dietary patterns were classified based on the Food Frequency Questionnaire (FFQ) using k-means clustering. Specifically, children's dietary information was collected through an FFQ completed by their parents. The questionnaire included 38 items and covered various aspects of dietary behavior, such as the child's overall diet type (e.g., omnivorous or vegetarian), eating patterns (e.g., meal frequency, snacking habits, food refusal), frequency of beverage consumption (e.g., water, different types of milk, soy milk, soda, sports drinks, and juice), and intake of specific food groups including dairy products (e.g., cheese and yogurt), fruits, vegetables, meats, and eggs. Parental responses to the FFQ were used as input features for each participant, and k-means clustering was performed to identify dietary patterns. The optimal number of clusters (k = 3) was determined using the elbow method and explained approximately 20% of the total variance in dietary intake (Figure S3). As shown in the radar plot (Figure S4), cluster 1 was characterized by relatively higher consumption frequencies of meat, snack foods, sugary beverages, and refined grain products and was therefore described as a meat and snack-rich pattern. Cluster 2 showed relatively higher consumption of dairy-related foods, including milk and yogurt products, and was described as a dairy-rich pattern. Cluster 3 was characterized by relatively higher consumption of fish/seafood and legumes and was described as a seafood-rich pattern. The identified three clusters were encoded as a three-category variable and used as a dietary pattern covariate.

### Statistical analysis

Descriptive characteristics were expressed as mean ± standard deviation (SD) or median with interquartile range (IQR) for continuous variables and as frequencies with percentages for categorical variables. We applied the Multivariate Association with Linear Models (MaAsLin3) framework to screen for associations between plasma and stool HFR concentrations and the relative abundance of gut microbial species and functional pathways, with each exposure–outcome pair analyzed separately. Functional pathways were derived from metatranscriptomic data. Prior to modeling, the HFR concentrations in plasma and stools were log-transformed. The relative abundance of species and functional pathways was transformed using a pseudo-log procedure in which zeros were replaced with half of the minimum non-zero value in the feature, and the resulting values were log2-transformed to reduce skewness and stabilize variance. Models were adjusted for covariates, including the child's sex, family income, parents' education level, and dietary patterns derived from FFQ, and samples' sequencing depth. Models involving metatranscriptomic functional pathways were additionally adjusted for sequencing batch to account for the observed batch effect. The detailed settings of the MaAsLin3 model were provided in Table S1. False discovery rate (FDR) correction using the Benjamini–Hochberg method was applied separately for each HFR compound across all tested microbial species and, separately, across all tested functional pathways. Associations with an FDR < 0.2 were treated as significant associations, consistent with prior microbiome studies.^52^ ^,^ ^53^

To evaluate the robustness of our findings, a series of sensitivity analyzes were performed for both species- and pathway-level association analyzes. First, the main models assessing associations of HFR exposures with gut microbial species and pathways were additionally and individually adjusted for body mass index (BMI) and antibiotic use in the past year (yes vs. no), both known predictors of the childhood gut microbiome. Second, instead of adjusting for overall dietary patterns, sensitivity analyzes were performed by additionally adjusting the main models for specific dietary factors. These included a fiber-related food frequency (i.e., sum of the consumption frequencies of fruits, vegetables, legumes, bread/cereals, and pasta/rice), given the established role of dietary fiber in shaping the gut microbiome,^54^ and the sum of the frequencies of HFR-relevant animal-derived food frequency (i.e., whole milk, cheese, yogurt, red meat, and fish/seafood), given their potential contribution to dietary HFR exposure.^18^ ^,^ ^55^ Third, both prevalence- and abundance-based filtering were applied by treating relative abundance values below 0.01% as undetected, re-applying the 10% prevalence filter, and re-running the main models to assess the influence of low-abundance features on the results. Fourth, the time interval (days) between blood and stool sample collection was additionally adjusted for in the main models to assess the potential influence of temporal variation in sample collection on the results.

We further performed targeted cross-omics mediation analyzes that integrated metagenomic species-level and metatranscriptomic pathway-level findings to examine whether microbial species mediated associations between HFR exposures and microbial functional pathways. We first identified species–pathway pairs that were both significantly associated with the same HFR compound in the MaAsLin3 models. For these candidate pairs, we further examined the associations between species and pathway relative abundance using Spearman's partial correlation while adjusting for the same covariates as in the main models. Only species–pathway pairs that remained significantly correlated were carried forward to the mediation analysis. The mediation models were constructed using the mediation R package, estimating average causal mediation effects (ACME) based on 1000 bootstrap simulations. The proportion mediated, defined as the percentage of the total exposure–outcome association explained by the mediator, was also calculated. To evaluate the directionality and robustness of the mediation effects, we conducted reverse mediation analyzes by swapping the mediator and outcome, i.e., testing models with microbial pathways as the mediators and species as the outcomes. Mediation pairs that yielded significant ACME (*p* < 0.05) in the reverse direction were considered less likely to reflect a biologically plausible pathway and were excluded from interpretation. All models were adjusted for the same set of covariates as in the main association analyzes.

All statistical analyzes were completed using R (version 4.1.0; R Development Core Team). A two-tailed *p* less than 0.05 or FDR less than 0.2 was considered statistically significant.

## Results

### Population characteristics

A total of 182 children aged 8–12 y were included in this study. All participants had stool HFR measurements, and 140 also had plasma HFR measurements. Among the 182 children, the average age was 11 (SD: 1.2) y old, and 46.2% of them were identified as male. The average annual family income was approximately 106,632 Canadian dollars (SD: 77,812), and over half of their parents reported having attained a college or university education. Regarding dietary behavior, 18.1%, 35.7%, and 46.2% of the children were categorized into meat and snack-rich pattern, Dairy-rich pattern, and Seafood-rich pattern, respectively. The summary statistics of each food group consumption frequency were provided in Table S2. The average BMI of the participants was 18.33 (SD: 3.12). The demographic characteristics of children with plasma HFR data were similar to those of the overall study population (Table 1).

**Table 1.** Characteristic of population.

Characteristics	Stool set (*n* = 182)	Plasma set (*n* = 140)
Age (years [mean (SD)])	11.0 (1.2)	10.9 (1.2)
**Sex (%)**		
Male	84 (46.2)	60 (42.9)
Female	98 (53.8)	80 (57.1)
Family income (k CAD [mean (SD)])	106.63 (77.81)	114.99 (72.91)
**Parents' education level (%)**		
Secondary	8 (4.4)	5 (3.5)
College	30 (16.5)	26 (18.6)
University	90 (49.4)	67 (47.9)
Vocational training	54 (29.7)	42 (30.0)
**Food frequency questionnaire cluster**		
Meat and snack-rich pattern	33 (18.1)	20 (14.3)
Dairy-rich pattern	65 (35.7)	47 (33.6)
Seafood-rich pattern	84 (46.2)	73 (52.1)
BMI	18.33 (3.12)	18.35 (3.10)
**Antibiotic usage in the past year**		
Yes	27 (14.8)	26 (18.6)
No	155 (85.2)	114 (81.4)
Metagenomic sequencing depth {M reads [mean (SD)]}	35.26 (27.44)	35.15 (27.44)
Metatranscriptomic sequencing depth {M reads [mean (SD)]}	33.98 (31.47)	35.02 (33.13)
**HFR Concentrations {ng/g dw/lipid [Median (IQR)]}** *		
BDE-47	0.57 [0.15, 3.53]	4.82 [2.22, 12.93]
BDE-99	0.09 [0.05, 0.29]	1.82 [0.96, 4.81]
BDE100	0.03 [0.01, 0.09]	1.26 [0.38, 2.32]
BDE-153	0.04 [0.02, 0.06]	4.32 [2.13, 7.03]
BB-153	–	0.16 [0.11, 0.60]
BDE-209	18.83 [10.07, 68.52]	–
syn-DP	0.02 [0.01, 0.03]	–
anti-DP	0.09 [0.06, 0.16]	–
Dec602	0.003 [0.002, 0.005]	–
Dec603	0.001 [0.001, 0.003]	–

Note: CAD, Canadian dollar. SD, standard deviation. HFR, Halogenated Flame Retardant. IQR, Interquartile Range.

^*^ The units of HFR concentrations in stool and plasma samples are ng/g dry weight (dw) and ng/g lipid, respectively.

HFR concentrations and detection rates in children's plasma and stool samples were summarized in Table 1 and Table S3, respectively. BDE-47, BDE-99, BDE-100, BDE-153, and BB-153 were detected in 52.9%–97.9% of the plasma samples, with the median concentration ranging from 0.16 ng/g lipid to 4.82 ng/g lipid. Compounds of HFRs, including BDE-47, BDE-99, BDE-100, BDE-153, BDE-209, syn-DP, and anti-DP were detected in 53.0%–91.8% of the stool samples, with the highest median concentrations of 18.83 g/g dw for BDE-209, followed by 0.57 ng/g dw for BDE-47. In addition, we also included Dec602 and Dec603 that were detected in 36.1% and 38.8% of the stool samples.

### Associations between HFR exposures and gut microbial species

Metagenomic sequencing identified 244 bacterial species that appeared in at least 10% of the study population. The relative abundance of the dominant species across samples is shown in Figure S5, with relative abundance and prevalence information for all detected microbial species provided in Table S4. Using MaAsLin3 models adjusted for covariates, we identified seven significant associations of children's plasma and stool HFR concentrations with the relative abundance of gut microbial species (q-values < 0.2) (Figure 2A, Table S5). A one–log-unit increase in children’s plasma BDE-47 and BDE-99 concentrations was significantly associated with lower relative abundances of *Clostridium sp. CAG:221* (*β* = −0.93, 95% CI: −1.41, −0.45, q-value = 0.197) and *Bacteroides dorei* (*β* = −1.37, 95% CI: −2.06, −0.69, q-value = 0.168), respectively. Among the stool-measured HFRs, BDE-153 showed significant negative associations with *Eubacterium. sp. CAG:38* (*β* = −0.64, 95% CI: −1.07, −0.21, q-value = 0.196) and *Roseburia intestinalis* (*β* = −0.74, 95% CI: −1.24, −0.25, q-value = 0.196), both were recognized as short-chain fatty acid–producing taxa.^56^ ^,^ ^57^ In addition, significant positive associations were observed between stool BDE-153 and the relative abundance of *Faecalibacterium sp. CAG:74* (*β* = 0.54, 95% CI: 0.22, 0.87, q-value = 0.188), *Faecalibacterium sp. CAG:74_58_120* (*β* = 0.73, 95% CI: 0.38, 1.08, q-value = 0.072), and *Alistipes putredinis* (*β* = 0.49, 95% CI: 0.21, 0.76, q-value = 0.182), taxa previously implicated in gut immune regulation and anti-inflammatory processes.^58^ These significantly associated species were relatively common in the study population, with prevalence ranging from 52.86% to 93.96% (Table S6).

### Associations between HFR exposures and gut microbial functional pathways

A total of 282 active functional pathways were identified by mapping shotgun metatranscriptomic reads to the MetaCyc database. Eight significant associations between stool HFR concentrations and gut microbial functional pathways were identified after FDR adjustment (*q-value* < 0.2) (Figure 2B–D, Table S7), while no significant association were identified between plasma HFR concentrations and functional pathways. Specifically, stool BDE-153 levels were positively associated with relative abundance of 4-aminobutanoate degradation V (*β* = 0.20, *q-value* = 0.057), superpathway of pyridoxal 5'-phosphate biosynthesis and salvage (*β* = 0.18, *q-value* = 0.091), tricarboxylic acid (TCA) cycle II (*β* = 1.01, *q-value* = 0.12), cytidine diphosphate-diacylglycerol (CDP-diacylglycerol) biosynthesis III (*β* = 0.30, *q-value* = 0.12), and 4−amino−2−methyl−5−diphosphomethyl-pyrimidine biosynthesis II (*β* = 0.14, *q-value* = 0.17), and negatively associated with myo-, chiro- and scyllo-inositol (*β* = −0.89, *q-value* = 0.057). Stool anti-DP concentrations were positively linked to 4-aminobutanoate degradation V (*β* = 0.33, *q-value* = 0.005), phosphatidylcholine acyl editing (*β* = 0.77, *q-value* = 0.014), and TCA cycle IV (*β* = 0.23, *q-value* = 0.050). Additional positive associations of stool anti-DP concentrations were observed with pathways involved in L-lysine biosynthesis and L-histidine degradation, along with an inverse association with 5-aminoimidazole ribonucleotide biosynthesis II. In addition, stool Dec603 concentrations were negatively associated with several functional pathways, including pathways related to sulfate assimilation, L-methionine biosynthesis, and phytol degradation. The identified pathways represent several microbial metabolic processes, including 4-aminobutanoate (*γ*-Aminobutyric acid, GABA) degradation, pyridoxal 5′-phosphate or vitamin B6 biosynthesis and salvage, central carbon metabolism through the TCA cycle, sulfate and amino acid metabolism, and membrane lipid-related pathways including CDP-diacylglycerol biosynthesis and phosphatidylcholine acyl editing, and inositol degradation.

### Sensitivity analyzes

Effect directions and magnitudes of the above species- and pathway-level associations were mainly unchanged after additional adjustment for children’s BMI and antibiotic use within the past year, although some associations showed attenuated statistical significance after FDR correction (Figures S6–S7, Tables S8–S11). Similarly, additional adjustment for fiber-related food frequency and HFR-relevant animal-derived food frequency yielded generally consistent effect directions and magnitudes, with two additional species, *Lachnospira pectinoschiza* and *Eubacterium sp. 192*, reaching statistical significance in association with stool BDE-153 (Figure S8; Tables S12-S13). Notably, *Lachnospira pectinoschiza* is a pectinolytic species associated with dietary fiber fermentation,^59^ broadly consistent with the functional characteristics of *Roseburia intestinalis* and *Eubacterium sp. 138*, which were negatively associated with stool BDE-153 in the primary analysis. Applying both abundance- and prevalence-based filtering by treating relative abundance values below 0.01% as undetected and re-running the main models didn't substantially change the results (Figure S9; Tables S14-S15). Adjustment for the time interval (days) between blood and stool sample collection also yielded generally consistent associations with the primary analysis, although one association no longer reached the FDR significance threshold (Figure S10; Tables S16-17).

**Figure 2.**
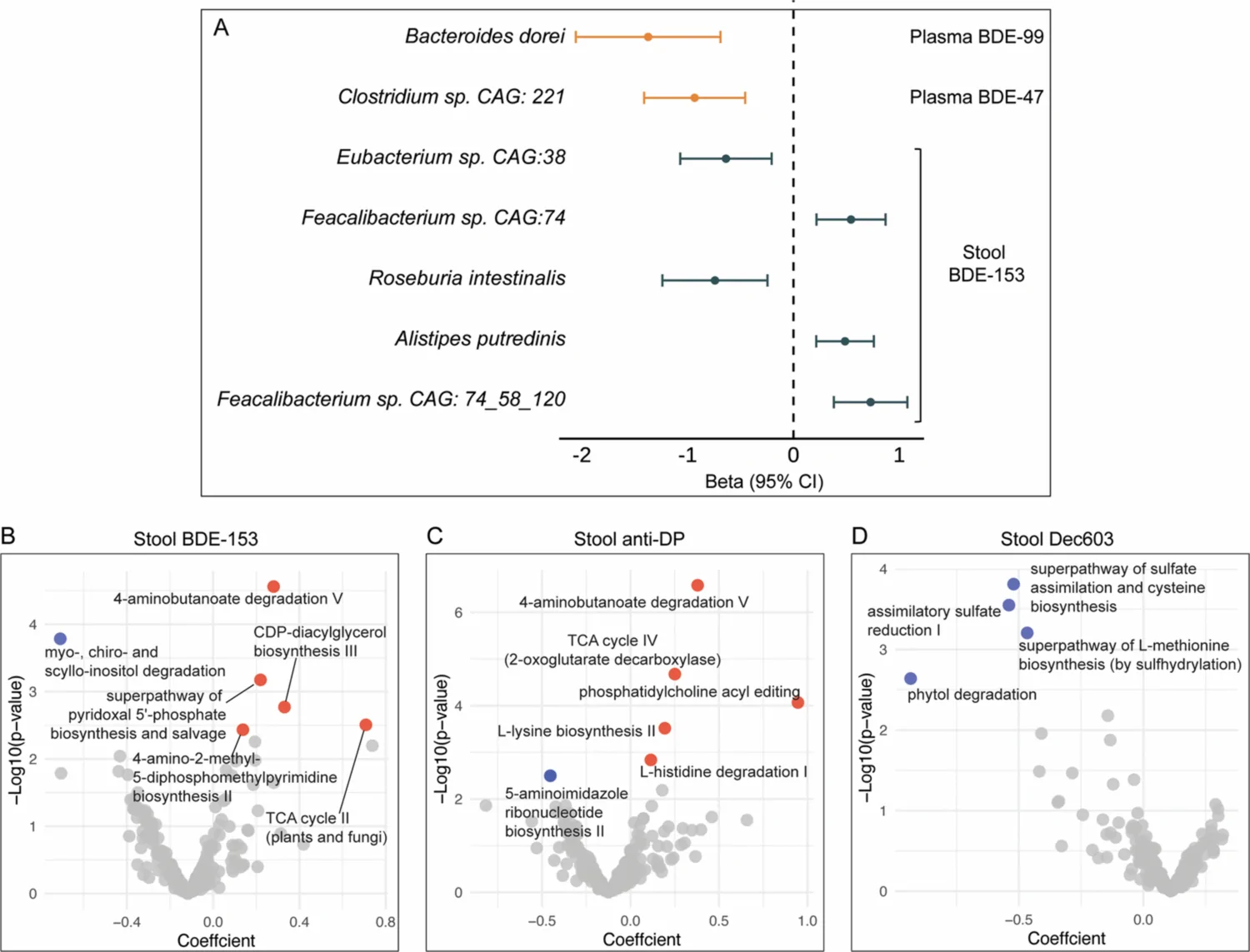
Associations of childhood plasma and stool HFR concentrations with the relative abundance of gut microbial species (A) and associations of childhood stool BDE-153 (B), anti-DP (C), and Dec603 (D) concentrations and the relative abundance of gut microbial functional pathways. Models were adjusted for the child's sex, family income, parents' educational levels, dietary patterns, and samples' sequencing depth at the 8- to 12-y-old follow-up. Models for associations between HFR concentrations and the relative abundance of functional pathways were additionally adjusted for sequencing batch to account for batch effects. Only significant associations between HFRs and species with an FDR q-value of <0.2 were presented. Pathways with an FDR q-value of <0.2 are labeled in color: blue dots represent negative associations, and red dots represent positive associations. All numeric results can be found in the Supplementary Table S5 and S7.

### Mediation analysis

Based on the MaAsLin3 results, five microbial species and six functional pathways were significantly associated with stool BDE-153 concentrations. Among these, four species–pathway pairs remained significantly correlated after covariate-adjusted partial Spearman correlation analyzes and were therefore carried forward as candidates for mediation analysis (Figure 3A, Table S18). Given the limited number of prespecified mediation tests, *p*-values were reported without additional FDR correction. Of these, the relative abundance of *Faecalibacterium* sp. *CAG:74_58_120* significantly mediated the positive association between stool BDE-153 concentration and the CDP-diacylglycerol biosynthesis III pathway. The estimated indirect effect was *β* = 0.042 (95% CI: 0.001 0.094; *p* = 0.036), corresponding to a proportion mediated of 27.0% (Figure 3B, Table S17-18). No other significant mediation effects were observed, and reverse mediation analyzes were not statistically significant for any of the tested paths (Figure 3C–E, Tables S18-19).

**Figure 3.**
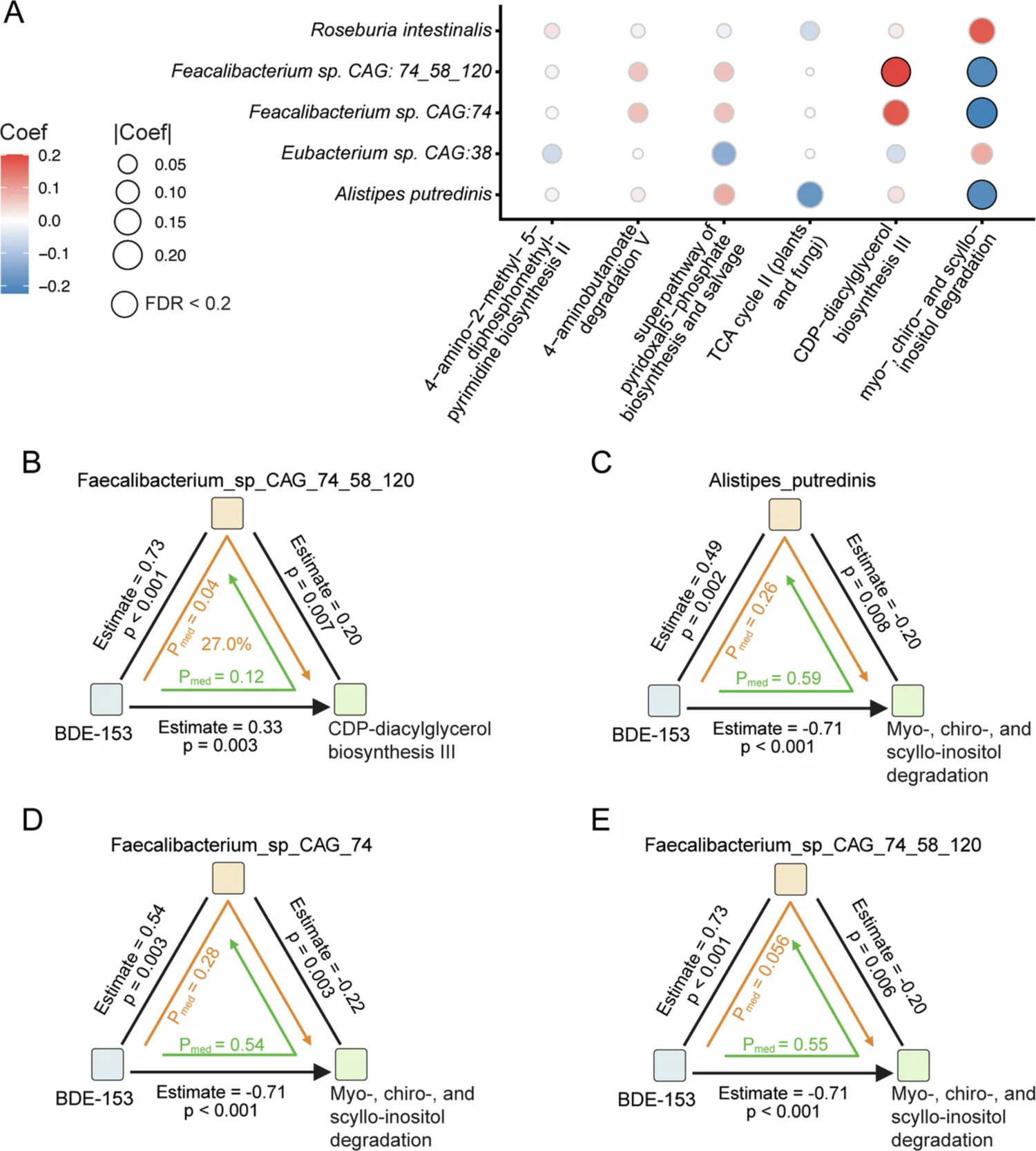
Mediation effects of species abundance on associations between BDE-153 and gut microbial functional pathways. (A) Spearman's partial correlations between species and functional pathways that were each significantly associated with BDE-153. (B) *Faecalibacterium sp. CAG: 74_58_120'*s mediation effect on the association between BDE-153 and CDP-diacylglycerol biosynthesis III. (C) *Alistipes putredinis*'s mediation effect on the association between BDE-153 and myo−, chiro−, and scyllo−inositol degradation. (D) *Faecalibacterium sp. CAG: 74'*s mediation effect on the association between BDE-153 and myo−, chiro−, and scyllo−inositol degradation. (E) *Faecalibacterium sp. CAG: 74_58_120'*s mediation effect on the association between BDE-153 and myo−, chiro−, and scyllo−inositol degradation. All numeric results can be found in Supplementary Table S18 and S19.

## Discussion

In this study, we explored associations of childhood plasma and stool HFR levels with the relative abundance of gut microbial species and active pathways in 8–12-year-old children in the GESTE cohort. To our knowledge, this is one of the first studies to integrate plasma- and stool-based HFR exposure assessment with shotgun metagenomic and metatranscriptomic profiling of the childhood gut microbiome. We identified seven gut microbial species and fifteen functional pathways whose relative abundance was associated with HFR concentrations. Through mediation analysis, we also provide evidence that *Faecalibacterium* species partially mediate the association between stool BDE-153 exposure and higher microbial functional shifts in phospholipid biosynthesis. Together, these findings suggest that HFR exposure may be associated with variations in the gut microbial ecosystem in school-aged children, involving both microbial community composition and functions.

To contextualize the observed HFR concentrations, we compared exposure levels in our study population with those reported in prior birth cohorts. Two well-characterized cohorts, the Center for the Health Assessment of Mothers and Children of Salinas (CHAMACOS) and the Health Outcomes and Measures of the Environment Study (HOME), have reported plasma HFR levels in children of similar age.^60^ ^,^ ^61^ The CHAMACOS cohort includes predominantly Mexican American children from an agricultural community in California's Salinas Valley,^61^ whereas the HOME cohort includes children from the greater Cincinnati, Ohio metropolitan area with a more socioeconomically diverse urban/suburban population.^60^ Overall, HFR levels in the GESTE cohort were substantially lower than those observed in CHAMACOS and HOME. For example, the median concentration of BDE-47 in plasma among GESTE children was 4.82 ng/g lipids, compared with higher levels reported in CHAMACOS, 35.2 ng/g lipids,^61^ and HOME, 18.3 ng/g lipids.^60^ These differences highlight variability in exposure levels across different populations and suggest that lower-level HFR exposures, such as those observed in the GESTE cohort, warrant investigation regarding their potential health effects.

At the species level, we found two significant associations of plasma BDE-99 and five significant associations of BDE-153 with gut microbial species. Some of these identified species have known links to gut function and human health. For example, *Bacteroides dorei* was negatively associated with plasma BDE-99, and *Eubacterium spp*. and *Roseburia intestinalis* were negatively associated with stool BDE-153. These taxa are well-known producers of short-chain fatty acids, which help regulate inflammation and support host energy metabolism.^56^ ^,^ ^57^ ^,^ ^62^ These negative associations might indicate the potential links between HFR exposure and fewer short-chain fatty acid (SCFA) producers in the gut. This finding is generally in line with conclusions from several previous studies. In a birth cohort of 267 mother–infant pairs from the Norwegian Microbiota Cohort, Iszatt et al. measured associations of breast milk PBDEs with infant gut microbial composition and SCFA profiles at 1 month of age. They found that higher PBDE-28 exposure was associated with lower fecal propionic acid levels, a key SCFA involved in host metabolism and immune regulation.^31^ Another previous *in vitro* study also observed that low-dose Decabromodiphenyl ethane, another HFR compound, reduced SCFA levels in a simulated in vitro human gut microbial ecosystem.^28^ Taken together, despite differences in study design, both existing evidence and our findings suggest that HFR exposure may disrupt SCFA-producing gut bacteria and consequently reduce microbial SCFA production. In addition, we identified a gut commensal, *Alistipes putredinis,* were positively associated with stool BDE-153 exposures. *Alistipes putredinis* has been implicated in maintaining metabolic health by supporting gut barrier integrity and suppressing inflammation-related signaling pathways.^58^ ^,^ ^63^ ^,^ ^64^ Its enrichment under HFR exposures may therefore reflect an adaptive anti-inflammatory response or a selective advantage of taxa capable of tolerating hydrophobic chemical stress in the gut.

From the metatranscriptomic analyzes, no significant associations were identified between plasma HFR concentrations and gut microbial functional pathways. This may partly reflect the smaller plasma HFR analysis set and reduced statistical power. In addition, plasma HFR concentrations may reflect systemic exposure^65^ rather than direct exposure within the intestinal lumen. HFRs present in the intestinal lumen can directly interact with gut microbes and may therefore be more closely related to microbial functional pathway profiles. In contrast, plasma HFRs may influence the gut microbiome more indirectly through host-mediated mechanisms, such HFRs might instead influence the gut microbiome indirectly through host-mediated mechanisms, such as hepatic metabolism, bile acid signaling, and inflammatory responses.^66^ Therefore, gut microbial functional activities might be more sensitive to stool HFR concentrations.

We observed that stool concentrations of BDE-153, anti-DP, and Dec603 were associated with the activation of multiple functional pathways that can be broadly grouped into several categories: neurotransmitter metabolism (e.g., 4-aminobutanoate degradation), energy metabolism (e.g., TCA cycle), lipid/cofactor metabolism (e.g., CDP-diacylglycerol biosynthesis, phosphatidylcholine acyl editing), and sulfur metabolism (e.g., assimilatory sulfate reduction I). Several *in vitro* and *in vivo* studies had explored the effects of PBDE congeners and DP congeners on host gut microbial functional pathways and metabolomic response. In an in vitro system, using metatranscriptomic profiling, Defois et al. observed increased expression of gut microbial functional pathways related to lipid metabolism processes and plasma membrane following exposure to brominated flame retardants.^27^ Despite the lack of metatranscriptomic data, two animal studies have reported that exposure to BDE-47 disturbed host metabolic profiles that were correlated with gut microbiome species alterations. Specifically, Qiu et al. and Gao et al. found in rat models that maternal exposure to BDE-47 led to marked reductions in multiple fatty acids and TCA cycle intermediates, suggesting impairment of energy metabolism and lipolysis.^10^ ^,^ ^12^ Similarly, in the current study, we observed that exposure to BDE-153 and anti-DP was associated with higher activity of TCA cycles and phospholipid homeostasis-related microbial pathways, including CDP-diacylglycerol biosynthesis, a central precursor in microbial phospholipid synthesis,^67^ and phosphatidylcholine acyl editing, a lipid editing process that maintains membrane integrity and fluidity.^68^ It has been proposed that lipophilic compounds can increase membrane fluidity when crossing membranes, and bacterial cells counteract this by enhancing membrane rigidity through greater lipid saturation to prevent their intracellular accumulation.^69^ Thus, increased activity in these pathways may reflect an adaptive mechanism of bacteria to remodel the cell membrane to limit pollutant penetration or compensate for membrane perturbation. Additionally, a study also showed that CDP-diacylglycerol could regulate host lipid droplet growth and had potential implications in metabolic health.^70^ However, the links between activity of these phospholipid homeostasis–related pathways in the gut and host health remain unclear and warrant further investigation.

In addition, we also found that both stool BDE-153 and anti-DP were linked to higher activity of 4-aminobutanoate (*γ*-Aminobutyric acid, GABA) degradation in the gut microbiome. GABA is an essential neurotransmitter in the human central neuro system, and its local effects in the gut include neurotransmission via myenteric neurons, endocrine signaling by mucosal cells, and regulation of gut barrier integrity and immune activity.^71^ An increased abundance of the gut microbial GABA-degradation pathway has also been reported in children with tic disorders.^72^ The enrichment of this pathway under BDE-153 and anti-DP exposure might suggest a potential reduction in gut GABA availability, which might weaken gut–brain communication, promote intestinal inflammation, and impair epithelial barrier integrity.

Microbial sulfur metabolism was another functional process that were observed to be associated with HFR exposure. Specifically, Dec603 was negatively associated with several interconnected pathways involved in assimilatory sulfate reduction, sulfate assimilation and cysteine biosynthesis, and L-methionine biosynthesis by sulfhydrylation. These pathways constitute key components of microbial assimilatory sulfur metabolism, through which inorganic sulfate is reduced and incorporated into sulfur-containing amino acids such as cysteine and methionine. Experimental evidence suggests that disruption of bacterial assimilatory sulfate reduction can alter the routing of sulfur metabolism in the gut, with downstream effects on higher H_2_S production and intestinal inflammatory responses. Luo et al. showed that deletion of *cysJ*, a key gene involved in assimilatory sulfate reduction, impaired sulfate utilization but enhanced H_2_S production from L-cysteine degradation in *E. coli*.^73^ In mice, colonization with the *cysJ*-deficient strain resulted in elevated serum H_2_S and fecal sulfite levels and more severe intestinal inflammation.^73^ Although H_2_S and other sulfur metabolites were not measured in our study, the coordinated negative associations of Dec603 with multiple components of assimilatory sulfur metabolism raise the possibility that Dec603 exposure may be associated with varied microbial sulfur metabolism.

Through the mediation analysis, we found that higher abundance of *Feacalibacterium sp. CAG:74_58_120* significantly mediated the positive associations between BDE-153 and functional pathway CDP-diacylglycerol biosynthesis. To date, no published study has reported a mediating role of a specific microbial taxon, such as Faecalibacterium sp. CAG:74_58_120, in the association between HFR exposure and the CDP-diacylglycerol biosynthesis pathway. Nevertheless, findings from a large human metagenomic study by Chen et al. revealed a strong correlation between *Faecalibacterium prausnitzii*, a close relative of *Faecalibacterium sp. CAG:74_58_120*, and CDP-diacylglycerol biosynthesis, supporting our observed correlations between *Faecalibacterium spp.* and this pathway.^74^ The mediation results might suggest that the association between BDE-153 and higher transcription of microbial phospholipid-synthesis pathways might be partly mediated by *Faecalibacterium spp*. However, because of the cross-sectional design, these results should be interpreted as exploratory and hypothesis-generating rather than as evidence of causal relationships among HFR exposure, microbial species, and functional pathways. Longitudinal or experimental studies are needed to confirm the directionality of these associations.

Strengths of this study include the integration of cross-omics data, linking HFR exposure with both taxonomic and active functional profiles of the gut microbiome. The use of metagenomic and metatranscriptomic sequencing enabled high-resolution profiling of gut microbial communities, capturing both taxonomic composition and actively expressed functional pathways. The measurement of HFRs in both plasma and stool provided a more comprehensive assessment of internal exposure. Additionally, the targeted cross-omics mediation analysis allowed us to evaluate exposure–microbiome–function links, providing new insights into potential exposure–microbiome interactions. Finally, our study population is homogeneous in socioeconomic status and race, which minimizes residual confounding from socioeconomic factors and enhances the internal validity of our findings.

However, several limitations should be acknowledged. First, the cross-sectional design prevents us from inferring causal relationships between HFR exposure and gut microbiome features, including the possibility of reverse causality. However, evidence for bacterial transformation of HFRs has primarily been reported in environmental bacteria or enrichment cultures, including *Dehalococcoides*-containing enrichment cultures,^75^ *Sulfurospirillum*,^75^ *Stenotrophomonas*,^76^ and *Pseudomonas*.^77^ These taxa were not detected among the gut microbial species in our study population. Therefore, our data do not support reverse causality through known HFR-transforming bacteria. Second, the relatively modest sample size may have limited statistical power, particularly for detecting associations with smaller effect sizes after correction for multiple testing. However, multi-omics microbiome studies typically involve smaller cohorts due to the high cost of shotgun metagenomics and metatranscriptomics, and our sample size is comparable to or larger than many published studies using similar approaches.^78‐81^ Third, the average interval between blood and stool sample collection was 21 d. Because gut microbiome profiles may vary over short periods in children, this gap may have introduced non-differential measurement error in the microbiome assessment, potentially increasing uncertainty in the estimated associations.^82^ Fourth, while key covariates were adjusted for, the possibility of residual confounding from unmeasured factors such as household chemical exposure cannot be fully excluded. In addition, dietary intake was reported by parents, and some foods may have been consumed outside the home at this age, which may have introduced measurement error in the assessment of dietary covariates. Fifth, the absence of host metabolomics data may limit our ability to directly link microbial functional pathway alterations to downstream host biological processes. Finally, the sociodemographic characteristics of the GESTE cohort and the relatively low levels of HFR exposure may limit the generalizability of the identified associations to populations with different sociodemographic characteristics, ethnic compositions, or exposure profiles. The clinical relevance of the observed microbial variation also remains uncertain because its implications for host health have not yet been established. Future multi-omics studies integrating measurements of HFR metabolites, gut microbial metabolomic profiles, and downstream health outcomes across diverse populations would provide a more complete picture of the interactions between HFRs and the gut microbiome and their clinical implications.

In conclusion, among children from the GESTE cohort, plasma and stool concentrations of HFRs were associated with alterations in gut microbiome composition and function, characterized by lower abundance of SCFAs-producing species, higher activity of microbial pathways related to phospholipid homeostasis, GABA degradation, and lower levels of sulfate metabolism. A higher abundance of *Faecalibacterium sp. CAG:74_58_120* partially explained the positive association between stool BDE-153 and the CDP-diacylglycerol biosynthesis pathway. Future studies may investigate whether these variations in gut microbial community and function are related to children's health outcomes.

## Supplementary Material

FigureS6.tif FigureS6.tif

FigureS8.tif FigureS8.tif

Supplemental Materials_1.docx Supplemental Materials_1.docx

FigureS7.tif FigureS7.tif

FigureS5.tif FigureS5.tif

FigureS2.tif FigureS2.tif

FigureS9.tif FigureS9.tif

FigureS10.tif FigureS10.tif

FigureS4.tif FigureS4.tif

FigureS3.tif FigureS3.tif

FigureS1.tif FigureS1.tif

Supplemental Materials_2.xlsx Supplemental Materials_2.xlsx

Supplemental Materials_3_STORMS checklist_0917.docx Supplemental Materials_3_STORMS checklist_0917.docx

Supplemental Materials_2.docx Supplemental Materials_2.docx

## Data Availability

The participants of this study did not give written consent for their data to be shared publicly, so due to the sensitive nature of the research, supporting data is not available. The data supporting the findings of this study are available upon request and upon review by GESTE Cohort staff.

## Code availability statement

Deidentified code used for the main analyzes and figure generation is available through the following GitHub repository: https://github.com/YidanZhang-YZ/HFR_GutMicrobiome.
