# Supplementary material for: Halogenated flame retardant exposure was associated with pediatric gut microbiome profiles in a Canadian cohort
Source: Gut Microbes Rep. 2026 Oct 18;3(1):2747517. doi: 10.1080/29933935.2026.2747517 (PMC13682408; doi:10.1080/29933935.2026.2747517)
Supplement: Supplementary file 1 [file KGMR_A_2747517_SM8557.docx]

**Supplemental Material 1**

**Halogenated flame retardant exposure was associated with pediatric gut microbiome profiles in a Canadian cohort**

Yidan Zhang, Feng Gao, Peng Gao, Yihan Fan, Xinlei Wang, Yuqiu Yang, Anthony Gagnon, Guillaume Martinez, Virginie Gillet, Jiping Zhu, Jean-Philippe Bellenger, Andrea Baccarelli, Larissa Takser, Yike Shen*

**Supplemental Materials 2 is provided as an Excel file and is available through the following GitHub repository:** <https://github.com/YidanZhang-YZ/HFR_GutMicrobiome>

**Table of Contents**

**Table S1.** MaAsLin3 model settings.

**Table S2.** Summary statistics for FFQ food group consumption frequency scores

**Table S3.** The Limit of Detection (LOD) and Detection Rate of HFRs in children's plasma and stool samples.

**Table S4.** Relative abundance and prevalence of all detected microbial species (available in the Excel Supplemental Materials 2, at <https://github.com/YidanZhang-YZ/HFR_GutMicrobiome>).

**Table S5.** Associations of childhood plasma and stool concentrations with the relative abundance of gut microbial species (available in the Excel Supplemental Materials 2).

**Table S6.** Relative abundance and prevalence of microbial species significantly associated with HFR exposures.

**Table S7.** Associations of childhood plasma and stool concentrations with the relative abundance of gut microbial functional pathways (available in the Excel Supplemental Materials 2).

**Table S8.** Associations of childhood plasma and stool HFR exposures with the relative abundance of gut microbial species. Models were additionally adjusted for children’s BMI (available in the Excel Supplemental Materials 2).

**Table S9.** Associations of childhood plasma and stool HFR exposures with the relative abundance of gut microbial functional pathways. Models were additionally adjusted for children’s BMI (available in the Excel Supplemental Materials 2).

**Table S10.** Associations of childhood plasma and stool HFR exposures with the relative abundance of gut microbial species. Models were additionally adjusted for children’s antibiotic usage in the last year (yes vs. no) (available in the Excel Supplemental Materials 2).

**Table S11.** Associations of childhood plasma and stool HFR exposures with the relative abundance of gut microbial functional pathways. Models were additionally adjusted for children’s antibiotic usage in the last year (yes vs. no) (available in the Excel Supplemental Materials 2).

**Table S12.** Associations of childhood plasma and stool HFRs with the relative abundance of gut microbial species. Models were additionally adjusted for specific dietary factors, including a fiber-related food frequency score and an HFR-relevant animal-derived food frequency score (available in the Excel Supplemental Materials 2).

**Table S13.** Associations of childhood plasma and stool HFRs with the relative abundance of gut microbial functional pathways. Models were additionally adjusted for specific dietary factors, including a fiber-related food frequency score and an HFR-relevant animal-derived food frequency score (available in the Excel Supplemental Materials 2).

**Table S14.** Associations between HFR exposures and gut microbial species applying abundance- and prevalence-based filtering (available in the Excel Supplemental Materials 2).

**Table S15.** Associations between HFR exposures and gut microbial functional pathways applying abundance- and prevalence-based filtering (available in the Excel Supplemental Materials 2).

**Table S16.** Associations of childhood plasma HFR exposures with the relative abundance of gut microbial species. Models were additionally adjusted for time interval (days) between blood and stool sample collection (available in the Excel Supplemental Materials 2).

**Table S17.** Associations of childhood plasma HFRs with the relative abundance of gut microbial functional pathways. Models were additionally adjusted for time interval (days) between blood and stool sample collection (available in the Excel Supplemental Materials 2).

**Table S18.** Spearman partial correlations between childhood gut microbial species and pathways (available in the Excel Supplemental Materials 2).

**Table S19.** Mediation effects of species abundance on associations between stool BDE-153 exposure and gut microbial functional pathways and mediation effects of functional pathway abundance on associations between stool BDE-153 exposure and gut microbial species.

**Figure S1.** Flow diagram of sequencing quality control and read count retention for metagenomic (A) and metatranscriptomic sequencing (B).

**Figure S2.** Principal component analysis of microbial species and functional pathway profiles by sequencing batch

**Figure S3.** Elbow plot for selection of the number of dietary clusters based on Food Frequency Questionnaire data.

**Figure S4.** Radar plot of dietary pattern clusters identified using k-means clustering of Food Frequency Questionnaire-derived food group intakes.

**Figure S5.** Gut microbial species composition among children with stool and plasma HFR measurements.

**Figure S6.** Associations of childhood plasma BDE-47 (A), BDE-99 (B), and stool BDE-153 (C) with the relative abundance of gut microbial species and associations of childhood stool BDE-153 (D) and anti-DP (E) concentrations and the relative abundance of gut microbial functional pathways. Models were additionally adjusted for children’s BMI. Species and pathways with an FDR q-value of <0.2 are labeled in red/blue: blue dots represent negative associations, and red dots represent positive associations. All results can be found in Supplementary Table S8-9.

**Figure S7.** Associations of childhood plasma BDE-47 (A), BDE-99 (B), and stool BDE-153 (C) with the relative abundance of gut microbial species and associations of childhood stool BDE-153 (D) and anti-DP (E) concentrations and the relative abundance of gut microbial functional pathways. Models were additionally adjusted for children’s antibiotic usage in the last year (yes vs. no). Species and pathways with an FDR q-value of <0.2 are labeled in red/blue: blue dots represent negative associations, and red dots represent positive associations. All results can be found in Supplementary Table S10-11.

**Figure S8.** Associations of childhood plasma BDE-47 (A), BDE-99 (B), and stool BDE-153 (C) with the relative abundance of gut microbial species and associations of childhood stool BDE-153 (D) and anti-DP (E) concentrations with the relative abundance of gut microbial functional pathways. Models were additionally adjusted for specific dietary factors, including a fiber-related food frequency score and an HFR-relevant animal-derived food frequency score. Species and pathways with an FDR q-value of <0.2 are labeled in red/blue: blue dots represent negative associations, and red dots represent positive associations. All results can be found in Supplementary Tables S12–S13.

**Figure S9.** Associations between HFR exposures and gut microbial species and functional pathways applying abundance- and prevalence-based filtering. Associations of childhood plasma BDE-47 (A), BDE-99 (B), and stool BDE-153 (C) with the relative abundance of gut microbial species, and associations of childhood stool BDE-153 (D) and anti-DP (E) concentrations with the relative abundance of gut microbial functional pathways are shown. Relative abundance values below 0.01% were treated as undetected, the 10% prevalence filter was re-applied, and the main models were re-run. Species and pathways with an FDR q-value <0.2 are labeled in red/blue: blue dots represent negative associations, and red dots represent positive associations. All results are provided in Supplementary Tables S14–S15.

**Figure S10.** Associations of plasma BDE-47 (A) and BDE-99 (B) exposures with gut microbial species. Models were additionally adjusted for time interval (days) between blood and stool sample collection. Species with an FDR q-value <0.2 are labeled in red/blue: blue dots represent negative associations, and red dots represent positive associations. All results are provided in Supplementary Tables S16–S17.

Table S1. MaAsLin3 model settings

| Parameter | Setting |
| --- | --- |
| Analysis framework | MaAsLin3 |
| Fixed effects | Sex, family income, parents’ education, dietary pattern, sequencing depth. |
| Random effects | NONE |
| Normalization | NONE |
| Transformation | PLOG |
| Multiple testing correction | Benjamini–Hochberg FDR |
| Prevalence filter | ≥10% non-zero abundance |
| Significance threshold | q < 0.2 |

Table S2. Summary statistics for FFQ food group consumption frequency scores

|  | Stool HFR analyze set | | | Plasma HFR analyze set | | |
| --- | --- | --- | --- | --- | --- | --- |
| Food group | Median (IQR) | Min | Max | Median (IQR) | min | max |
| Water | 7 (6, 8) | 3 | 8 | 7 (7, 8) | 3 | 8 |
| Whole milk (3.25%) | 1 (1, 1) | 1 | 7 | 1 (1, 1) | 1 | 6 |
| 1%‚2% milk | 4 (1, 6) | 1 | 8 | 4 (1, 6) | 1 | 8 |
| Skim milk (0%) | 1 (1, 1) | 1 | 7 | 1 (1, 1) | 1 | 7 |
| Chocolate milk | 2 (1, 3) | 1 | 8 | 2 (1, 3) | 1 | 7 |
| Drinkable yogurt | 2 (1, 3) | 1 | 7 | 2 (1, 3) | 1 | 7 |
| Soy milk | 1 (1, 1) | 1 | 8 | 1 (1, 2) | 1 | 7 |
| Diet soda | 1 (1, 2) | 1 | 6 | 1 (1, 2) | 1 | 6 |
| Regular soda | 2 (1, 2) | 1 | 5 | 2 (1, 2) | 1 | 3 |
| Sports drinks | 2 (1, 2) | 1 | 5 | 2 (1, 2) | 1 | 5 |
| Tomato/vegetable juice | 1 (1, 2) | 1 | 6 | 1 (1, 2) | 1 | 6 |
| 100% fruit juice | 4 (2, 6) | 1 | 8 | 4 (2, 5) | 1 | 8 |
| Fruit drinks/punch | 1 (1, 2) | 1 | 6 | 1 (1, 2) | 1 | 6 |
| Other beverages | 1 (1, 1) | 1 | 8 | 1 (1, 1) | 1 | 6 |
| Cheese | 5 (4, 6) | 1 | 7 | 5 (4, 6) | 1 | 7 |
| Yogurt | 4 (2, 5) | 1 | 7 | 4 (3, 5) | 1 | 7 |
| Pudding-type dessert | 1 (1, 2) | 1 | 6 | 2 (1, 2) | 1 | 5 |
| Ice cream | 2 (2, 3) | 1 | 6 | 2 (2, 3) | 1 | 6 |
| Fruits excluding juice | 6 (5, 7) | 1 | 8 | 6 (5, 7) | 1 | 8 |
| Vegetables excluding potatoes | 6 (6, 7) | 3 | 8 | 6 (6, 7) | 3 | 8 |
| Potatoes | 3 (3, 4) | 1 | 8 | 3 (3, 4) | 1 | 8 |
| Poultry | 3 (3, 4) | 1 | 7 | 3 (3, 4) | 1 | 6 |
| Red meat | 3 (3, 4) | 1 | 7 | 3 (3, 4) | 1 | 6 |
| Eggs | 3 (3, 4) | 1 | 6 | 3 (3, 4) | 1 | 6 |
| Fish/seafood | 3 (2, 3) | 1 | 5 | 3 (2, 3) | 1 | 5 |
| Legumes | 2 (2, 3) | 1 | 6 | 2 (2, 3) | 1 | 6 |
| Bread | 6 (5, 6) | 2 | 8 | 6 (5, 6) | 3 | 8 |
| Breakfast cereals | 4 (3, 5) | 1 | 7 | 3 (3, 5) | 1 | 6 |
| Pasta/rice | 4 (3, 4) | 2 | 7 | 4 (3, 4) | 2 | 8 |
| Pastries/cookies/chips | 4 (3, 5) | 1 | 7 | 4 (3, 5) | 1 | 6 |

Note: FFQ frequency scores were coded as follows: 1 = never, 2 = less than once per week, 3 = 1–2 times per week, 4 = 3–4 times per week, 5 = 5–6 times per week, 6 = 1–2 times per day, 7 = 3–4 times per day, and 8 = 5 or more times per day.

Table S3. The Limit of Detection (LOD) and Detection Rate of HFRs in children's plasma and stool samples.

| HFRs | *N* | LOD | Detection (%) |
| --- | --- | --- | --- |
| Plasma sample (ng/g lipid) |  |  |  |
| BDE-47 | 140 | 1.534 | 85 |
| BDE-99 | 140 | 1.359 | 57.1 |
| BDE100 | 140 | 0.221 | 85 |
| BDE-153 | 140 | 0.531 | 97.9 |
| BB-153 | 140 | 0.152 | 52.9 |
| Stool sample (ng/g dw) |  |  |  |
| BDE-47 | 182 | 0.13 | 78.1 |
| BDE-99 | 181 | 0.07 | 53 |
| BDE-100 | 182 | 0.01 | 70.5 |
| BDE-153 | 182 | 0.01 | 83.6 |
| BDE-209 | 122 | 1.28 | 95.1 |
| syn-DP | 182 | 0.01 | 79.2 |
| anti-DP | 182 | 0.02 | 91.8 |
| Dec602 | 182 | 0.004 | 36.1 |
| Dec603 | 182 | 0.002 | 38.8 |

Note: HFRs, Halogenated flame retardants.

Table S6. Relative abundance and prevalence of microbial species significantly associated with HFR exposures.

| Species | Prevalence | Mean relative abundance | Median relative abundance |
| --- | --- | --- | --- |
| Clostridium_sp_CAG_221 | 68.57 | 0.15 | 0.03 |
| Bacteroides_dorei | 52.86 | 0.57 | 0.01 |
| Alistipes_putredinis | 93.96 | 1.47 | 1.30 |
| Faecalibacterium_sp_CAG_74 | 87.91 | 1.41 | 0.97 |
| Faecalibacterium_sp_CAG_74_58_120 | 85.71 | 0.13 | 0.05 |
| Roseburia_intestinalis | 70.88 | 0.87 | 0.08 |
| Eubacterium_sp_CAG_38 | 60.99 | 0.12 | 0.01 |

Table S19. Mediation effects of species abundance on associations between stool BDE-153 exposure and gut microbial functional pathways and mediation effects of functional pathway abundance on associations between stool BDE-153 exposure and gut microbial species

| Exposure | Mediator | Outcome | ACME | *p* | Proportion Mediated |
| --- | --- | --- | --- | --- | --- |
| Stool BDE153 | *Faecalibacterium_sp_*  *CAG_74_58_120* | PWY-7237 | -0.08 (-0.20, 0.001) | 0.056 | 54.4 |
| Stool BDE153 | *Faecalibacterium_sp_*  *CAG_74_58_120* | PWY-5981 | 0.04 (0.001, 0.10) | 0.036 | 27.0 |
| Stool BDE153 | *Alistipes_putredinis* | PWY-7237 | -0.03 (-0.11, 0.03) | 0.256 | 21.1 |
| Stool BDE153 | *Faecalibacterium_sp_*  *CAG_74* | PWY-7237 | -0.04 (-0.12, 0.02) | 0.278 | 26.4 |
| Stool BDE153 | PWY-7237 | *Faecalibacterium_sp_*  *CAG_74_58_120* | 0.03 (-0.06, 0.16) | 0.548 | 5.47 |
| Stool BDE153 | PWY-7237 | *Alistipes_putredinis* | 0.02 (-0.05, 0.07) | 0.588 | 3.51 |
| Stool BDE153 | PWY-7237 | *Faecalibacterium_sp_*  *CAG_74* | 0.04 (-0.07, 0.16) | 0.542 | 9.33 |
| Stool BDE153 | PWY-5981 | *Faecalibacterium_sp_*  *CAG_74_58_120* | 0.10 (-0.03, 0.27) | 0.116 | 16.84 |

Note: ACME, Average Causal Mediation Effect. PWY-7237: myo-, chiro- and scyllo-inositol degradation, PWY-5981: CDP-diacylglycerol biosynthesis III.

**Figure S1.** Flow diagram of sequencing quality control and read count retention for metagenomic (A) and metatranscriptomic sequencing (B).

**Figure S2.** Principal component analysis of microbial species and functional pathway profiles by sequencing batch

**Figure S3.** Elbow plot for selection of the number of dietary clusters based on Food Frequency Questionnaire data

**Figure S4.** Radar plot of dietary pattern clusters identified using k-means clustering of Food Frequency Questionnaire-derived food group intakes.

**Figure S5.** Gut microbial species composition among children with stool and plasma HFR measurements.

**Figure S6.** Associations of childhood plasma BDE-47 (A), BDE-99 (B), and stool BDE-153 (C) with the relative abundance of gut microbial species and associations of childhood stool BDE-153 (D) and anti-DP (E) concentrations and the relative abundance of gut microbial functional pathways. Models were additionally adjusted for children’s BMI. Species and pathways with an FDR q-value of <0.2 are labeled in red/blue: blue dots represent negative associations, and red dots represent positive associations. All results can be found in Supplementary Tables S8-9.

**Figure S7.** Associations of childhood plasma BDE-47 (A), BDE-99 (B), and stool BDE-153 (C) with the relative abundance of gut microbial species and associations of childhood stool BDE-153 (D) and anti-DP (E) concentrations and the relative abundance of gut microbial functional pathways. Models were additionally adjusted for children’s antibiotic usage in the last year (yes vs. no). Species and pathways with an FDR q-value of <0.2 are labeled in red/blue: blue dots represent negative associations, and red dots represent positive associations. All results can be found in Supplementary Table S10-11.

**Figure S8.** Associations of childhood plasma BDE-47 (A), BDE-99 (B), and stool BDE-153 (C) with the relative abundance of gut microbial species and associations of childhood stool BDE-153 (D) and anti-DP (E) concentrations with the relative abundance of gut microbial functional pathways. Models were additionally adjusted for specific dietary factors, including a fiber-related food frequency score and an HFR-relevant animal-derived food frequency score. Species and pathways with an FDR q-value of <0.2 are labeled in red/blue: blue dots represent negative associations, and red dots represent positive associations. All results can be found in Supplementary Tables S12–S13.

**Figure S9.** Associations between HFR exposures and gut microbial species and functional pathways applying abundance- and prevalence-based filtering. Associations of childhood plasma BDE-47 (A), BDE-99 (B), and stool BDE-153 (C) with the relative abundance of gut microbial species, and associations of childhood stool BDE-153 (D) and anti-DP (E) concentrations with the relative abundance of gut microbial functional pathways are shown. Relative abundance values below 0.01% were treated as undetected, the 10% prevalence filter was re-applied, and the main models were re-run. Species and pathways with an FDR q-value <0.2 are labeled in red/blue: blue dots represent negative associations, and red dots represent positive associations. All results are provided in Supplementary Tables S14–S15.

**Figure S10.** Associations of plasma BDE-47 (A) and BDE-99 (B) exposures with gut microbial species. Models were additionally adjusted for time interval (days) between blood and stool sample collection. Species with an FDR q-value <0.2 are labeled in red/blue: blue dots represent negative associations, and red dots represent positive associations. All results are provided in Supplementary Tables S16–S17.
