# Supplementary material for: Halogenated flame retardant exposure was associated with pediatric gut microbiome profiles in a Canadian cohort
Source: Gut Microbes Rep. 2026 Oct 18;3(1):2747517. doi: 10.1080/29933935.2026.2747517 (PMC13682408; doi:10.1080/29933935.2026.2747517)
Supplement: Supplementary file 2 [file KGMR_A_2747517_SM8565.docx]

**Supplemental Material 2**

**Halogenated flame retardant exposure was associated with pediatric gut microbiome profiles in a Canadian cohort**

Yidan Zhang, Feng Gao, Peng Gao, Yihan Fan, Xinlei Wang, Yuqiu Yang, Anthony Gagnon, Guillaume Martinez, Virginie Gillet, Jiping Zhu, Jean-Philippe Bellenger, Andrea Baccarelli, Larissa Takser, Yike Shen*

Supplemental Materials 2 is provided as an Excel file and is available through the following GitHub repository: <https://github.com/YidanZhang-YZ/HFR_GutMicrobiome>
